# The effectiveness of interventions targeting specific out-of-home food outlets: protocol for a systematic review

**DOI:** 10.1186/2046-4053-3-17

**Published:** 2014-02-24

**Authors:** Frances C Hillier-Brown, Helen J Moore, Amelia A Lake, Ashley J Adamson, Martin White, Jean Adams, Vera Araujo-Soares, Charles Abraham, Carolyn D Summerbell

**Affiliations:** 1 Fuse, UKCRC Centre for Translational Research in Public Health, Newcastle, UK; 2 School of Medicine, Pharmacy and Health, Durham University, Stockton-on-Tees, UK; 3 Institute of Health & Society, Newcastle University, Newcastle NE1 7RU, UK; 4 University of Exeter Medical School, Exeter, UK

## Abstract

**Background:**

Eating out of the home has been associated with higher intakes of energy and fat and lower micronutrient intakes, as well as the development of obesity. Out-of-home food outlets (OHFOs) and the foods obtained from these outlets are an ideal target for interventions to improve diet and tackle obesity. This systematic review will explore the evidence for the effectiveness of promoting healthy behaviour through interventions that modify food practices in specific OHFOs.

**Methods/Design:**

We will search the databases MEDLINE, EMBASE, CINAHL, PsycINFO, ASSIA and the NHS Economic Evaluation Database for studies that have evaluated interventions conducted in an OHFO that aim to promote healthier menu offerings. OHFOs which are not openly accessible to the general public and supermarkets will be excluded. Included study designs will be randomised controlled trials, non-randomised controlled trials, controlled before-after studies, interrupted time series studies and evaluations of single interventions where outcome measures were assessed at least once pre and post-intervention (repeated measures studies).

**Discussion:**

This systematic review aims to synthesise the available evidence with regard to interventions that aim to change specific OHFOs in order to promote healthier menu offerings. The findings of this review will provide information on the types of interventions that have been evaluated and the context in which they are set, and provide insights into what interventions, and intervention functions, are most effective in different OHFO settings, along with any important innovation, implementation and cost implications.

The review has been registered with PROSPERO (registration no. CRD42013006931).

## Background

Eating a healthy diet can help reduce the risk of a range of non-communicable diseases and, alongside being physically active, protect against overweight and obesity [1]. Obesity is a global problem with relatively high prevalence in most high-income countries, although there is evidence that this is stabilising and even declining in some countries [2]. Although obesity prevalence is low in most low- and middle-income countries, the recent rapid rise in some of these countries has led to growing concern [3].

One of the drivers of obesity is excess energy intake. Therefore, changing dietary behaviours is central to tackling obesity [4]. Eating out of the home has been associated with higher intakes of energy and fat, and lower micronutrient intakes [5]. In particular, there is evidence that the consumption of take-away foods and fast-foods are determinants of excess weight gain [6]. The popularity and prevalence of eating out-of-home, including the consumption of take-away foods and fast-foods, has risen considerably over the last few decades. By the early 1990s eating out of the home had become ‘embedded’ in our culture [7] and, at the same time, fast food companies adopted policies of aggressive international expansion, leading to rapid global expansion most notably in the Asia Pacific, Middle East and Africa [8]. Out-of-home food outlets (OHFOs) and the foods obtained from these outlets are an ideal target for interventions to tackle obesity [9].

‘Out-of-home’ or ‘away-from-home’ foods are defined based on where the foods are obtained, rather than where they are eaten [10]. Out-of-home food tends to be ready-to-eat requiring no additional preparation. OHFOs are part of the wider food environment (also called the ‘foodscape’) [11,12]. OHFOs provide an opportunity to buy out-of-home, ready-to-eat, food or beverages for consumption on or off the premises. OHFOs do not include settings where food can be bought which needs any kind of preparation (even just heating up), for example from supermarkets or food stores. Nor do they include settings where food can be freely acquired (for example, allotments and gardens), or where food is given or donated as a gift (for example, foodbanks).

This systematic review will only focus on specific OHFOs. We will not include OHFOs which are not openly accessible to the general public, including those based in workplaces and educational institutions, or health or social care institutions. There is a relatively large body of research around workplace food environments [13] and educational food environments [14]. Similarly, supermarkets or food stores are a distinct type of food environment with a growing body of literature [15]; therefore, we will exclude interventions in supermarkets and food stores (but supermarket and food store cafes open to the public will be included).

While there is a wide range of research on food retailing in the UK and its association with dietary consumption and obesity [16], there is limited evidence on interventions that focus on promoting healthier menu offerings in the specific OHFOs as we have defined them above. In addition, and to our knowledge, there are no existing systematic reviews on this topic. This systematic review will explore the evidence for the effectiveness of promoting healthy dietary behaviour through interventions that modify food practices in specific OHFOs. This review is the first step within a programme of work which addresses the overarching research question of whether specific OHFO interventions can promote and provide healthier food choices for consumers while maintaining or improving retailers’ profits [17].

## Objectives

1. To identify, critically appraise, and summarise the relevant evidence (from repeated measures studies to RCTs) on the effectiveness and cost implications of interventions to promote healthier menu offerings in specific OHFOs.

2. From the studies identified in objective 1, to identify and summarise relevant evidence (including qualitative and quantitative data where available) describing the development and implementation, and process evaluations, of OHFO interventions. Information on consumer attitudes and preferences related to OHFO interventions identified in objective 1 will also be collated and summarised.

3. To identify and classify Behaviour Change Techniques (BCTs) used in interventions identified in objective 1.

## Methods

We will undertake a systematic review of effectiveness and cost implications using established methods, based on those used by the National Institute for Health and Care Excellence (NICE) [18], and will report the findings according to the PRISMA guidelines [19]. We will include evidence from repeated measures studies to RCTs that include a measure of change as an outcome of interest. The review has been registered with PROSPERO (registration number CRD42013006931).

### Population

The review will include studies conducted in any specific OHFO that serves, as its main business, ready-to-eat, prepared food for consumption on or off the premises. Specifically, in terms of setting, we will exclude OHFOs which are not openly accessible to the general public including those based in workplaces and educational institutions, or health or social care institutions. We will also exclude interventions in supermarkets and food stores (but supermarket and food store cafes will be included). The population of interest, therefore, will include customers of OHFOs (consumer level outcomes) as well as the businesses themselves (retail level outcomes).

### Interventions

The review will examine studies that have explored the effectiveness of any intervention that aims to change specific OHFOs in order to promote healthier menu offerings, including but not restricted to a reduction in energy intake. Interventions that focus exclusively on food hygiene or safety will be excluded. Examples of the types of interventions that we will expect to identify include those that focus on increasing the proportion of healthier food options available; promotions of healthier food options, food products or combinations; changing food formulation or cooking methods; and improving food and/or nutrition labelling.

### Comparator

Studies with and without comparators will be included in the review. There will be no restrictions on the type of comparator used in the study (for example, convenience comparison group, randomised control group, no intervention control and usual practice control).

### Outcomes

Outcomes of interest will include consumer outcomes and retail outcomes. Consumer outcomes will fall under the headings of behaviour (for example, dietary fat intake), purchasing (for example, sales of healthier menu choices), attitudes (for example, acceptance of intervention and attitude towards healthier menu choice) and preferences (for example, choice of healthier menu items over less healthy items). Retail outcomes will include changes in retail practices (for example, regular fat mayonnaise replaced with a lower fat version), process outcomes (for example, number of times a salt shaker is refilled) and profit. All outcomes reported that fall into any of these categories will be explored in detail and results synthesised.

We will also identify and critically summarise relevant evidence (quantitative and qualitative, including process evaluations) on the development and implementation of the included OHFO interventions in order to capture recent innovation in intervention design and inform future intervention development. Further analysis of the interventions identified will be undertaken to identify the BCTs of the underlying processes that were changed as well as the techniques or strategies used to change them [20]. We will also identify the modes of delivery, the intervention function (for example, environmental restructuring, incentivisation, training, education, and so on), and, if applicable, the policy category (for example, guidelines, regulation, legislation) in which the intervention fits. We will search for and identify a range of models/classifications of intervention modalities and choose one to use in the analysis alongside the BCT framework.

### Study designs

We will systematically search electronic databases for randomised controlled trials (RCTs), non-randomised controlled trials (NRCTs), controlled before-after studies (CBAs) and interrupted time series (ITS) studies. To the best of our knowledge, there is limited evidence from interventions of interest for this review from these types of study designs. Therefore, we propose to also include repeated measures studies and case series that have measured the experimental unit (for example, a group of people; the quantity of certain foods sold) before and after a single intervention; these studies do not include a control group.

### Literature searches

We will search the databases MEDLINE (Ovid), EMBASE (Ovid), CINAHL (Ebscohost), PsycINFO (Ebscohost), ASSIA (ProQuest) and the NHS Economic Evaluation Database (Wiley Cochrane). In an attempt to locate studies of relevance to the current ‘foodscape’ and the global expansion of fast food and out-of-home eating culture [8], we will limit our searches to studies published in the last 20 years (from 1993 to current). Searches will be limited to articles written in the English language. An example search strategy in MEDLINE is attached as an Additional file 1. In order to ensure adequate sensitivity of the search strategy, HM has piloted the search in MEDLINE (searched 24 October 2013). This resulted in 2,261 hits, of which all five key indicator papers [21-25] that we had identified from our knowledge prior to running the search, were included.

We will also contact known topic experts from a range of countries and send enquiry Email messages, seeking information about published, unpublished and ongoing interventions that could be included in our review. We plan to include a separate list of ongoing studies as part of the review, which could be included in future updates of the review.

### Data extraction and quality appraisal

Initial screening of titles and abstracts retrieved from the searches will be conducted by one reviewer (FH) with a random 10% of the sample independently screened by a second reviewer (AL). Full papers/reports of potentially relevant publications will be located and independently appraised by two researchers (FH and AL) to select those meeting the inclusion criteria. Data extraction and quality assessment of all included papers/reports will be conducted independently by two reviewers (FH and AL). Throughout, any discrepancies will be resolved through discussion with a third reviewer (CS). An electronic data extraction form has been developed and piloted to ensure consistency in data extraction between reviewers. Data to be extracted will include details of the project (aims, settings targeted, intervention description, resources, date started, date completed, study sponsor or funding source, and so on), study details (study design, population targeted, demographics, recruitment and follow-up rates, and so on), implementation information (context, experience of planners/implementers, collaboration, delivery fidelity, and so on), outcomes measured (and methods), and results. The quality of quantitative studies will be assessed using the Effective Public Health Practice Project Quality Assessment Tool for Quantitative Studies [26] as recommended by the Cochrane Public Health Review Group [27], and the Evaluation Tool for Qualitative Studies [28] will be used to assess the quality of qualitative studies.

### Analysis and synthesis

We will analyse the results using established methods, based on those used by the National Institute for Health and Care Excellence (NICE) [18]. Narrative synthesis will be conducted following the Economic and Social Research Council (ESRC) Narrative Synthesis Guidance [29]. Interventions will be grouped according to their intervention function and policy category, as well as the type of OHFO in which they are set. Any qualitative data will be analysed and synthesised thematically. We plan to match qualitative findings with quantitative findings using cross study synthesis as suggested by the Evidence for Policy and Practice Information and Co-ordinating Centre [30]. We will report our findings in accordance with PRISMA guidelines [19].

## Discussion

The review aims to summarise the evidence-base on the effectiveness and cost implications of interventions to promote healthier menu offerings in specific OHFOs. This will include collating information on development and implementation, process evaluations, and consumer attitudes and preferences, and the classification of the BCTs used. Our scoping search indicates that the volume of hits generated will be manageable and that the search strategy is adequately sensitive to identify relevant studies. A thorough search of the international grey literature evidence base is beyond the scope of this review; however, additional work is being carried out by the authors that will include an in-depth and systematic assessment of the grey literature evidence on the effectiveness and cost implications of interventions conducted in England to promote healthier menu offerings in specific OHFOs.

The method used for this review will allow the exploration of the types of interventions that have been evaluated and the context in which they are set, and provide insights into what interventions, and intervention functions, are most effective in different OHFO settings, along with any important innovation, implementation and cost implications. The findings from this review will be used to inform the development of future OHFO interventions, as well as policy and future practice.

## Abbreviations

OHFO: Out-of-home food outlet; RCT: Randomised controlled trial; NRCT: non-randomised controlled trial; CBA: controlled before-after study; ITS: interrupted time series.

## Competing interests

The authors declare that they have no competing interests.

## Authors’ contributions

FH assisted in the study design and development of methods, and drafted the manuscript. HM assisted in the conception of the study idea, the study design, the development of methods and the management of the study. AL assisted in the conception of the study idea, the study design and the development of methods, and helped to draft the manuscript. AA and MW assisted in the conception of the study idea and the study design, and have provided critical comments on drafts of the manuscript. JA, VA and CA assisted in the development of methods and have provided critical comments on drafts of the manuscript. CS assisted in the conception of the study idea, the study design, the development of methods and the management of the study, and helped to draft the manuscript. All authors have read and approved the final manuscript.

## Supplementary Material

Additional file 1 Example search strategy. Click here for file
