# Supplementary material for: The effectiveness of interventions targeting specific out-of-home food outlets: protocol for a systematic review
Source: Syst Rev. 2014 Feb 24;3:17. doi: 10.1186/2046-4053-3-17 (PMC3936835; doi:10.1186/2046-4053-3-17)
Supplement: Additional file 1 — Example search strategy. [file 2046-4053-3-17-S1.docx]

**Additional file – Example Search Strategy**

Medline (OVID)

| 1 | (out of home adj (food or eat$)).ab,ti. |
| --- | --- |
| 2 | Food away from home.ab,ti. |
| 3 | Hot food outlet$.ab,ti. |
| 4 | Food outlet$.ab,ti. |
| 5 | (Retail food adj (environment$ or foodscape)).ab,ti. |
| 6 | (Consumer food adj (environment$ or foodscape)).ab,ti. |
| 7 | Choice architecture.ab,ti. |
| 8 | (Takeaway or take away or take out or eating out or carry out$ or carryout$).ab,ti. |
| 9 | exp Restaurants/ |
| 10 | (Restaurant$ or cafe$ or Pub or Pubs or Public house$ or Diner$ or Inn or Inns or Eater$ or Fast food or Coffee shop$ or Sandwich shop$).ab,ti. |
| 11 | 1 or 2 or 3 or 4 or 5 or 6 or 7 or 8 or 9 or 10 |
| 12 | (Health$ adj2 (menu$ or eating or recipe$ or product$ or food$ or drink$ or beverage$ or meal$ or snack$ or dinner$ or lunch$ or cooking or catering practice$ or nutri$ or choice$)).ab,ti. |
| 13 | ((Health$ or food or food environment$ or environment$ or well being or wellbeing or wellness or eating) adj (polic$ or strategy$ or trial$ or intervention$ or program$ or educat$ or campaign$)).ab,ti. |
| 14 | ((Health$ or food or nutri$ or well being or wellbeing or wellness) adj2 (Display$ or Advert$ or Promot$ or Marketing or Point of purchase or improv$)).ab,ti. |
| 15 | exp Health promotion/ |
| 16 | exp Health education/ |
| 17 | exp Preventive medicine/ |
| 18 | exp Nutrition policy/ |
| 19 | (Obesity adj prevent$).ab,ti. |
| 20 | Environmental health.ab,ti. |
| 21 | ((Reformulat$ or modify$ or adapt$) adj2 (recipe$ or product$ or food$ or meal$)).ab,ti. |
| 22 | ((Nutri$ or food or calorie or menu) adj (signposting or label$)).ab,ti. |
| 23 | Food availability.ab,ti. |
| 24 | (Food price$ or food pricing).ab,ti. |
| 25 | ((Nutrition or cooking) adj6 (training or class$ or skill$)).ab,ti. |
| 26 | (Sugar or Salt or Fat or Fruit$ or Vegetable$).ab,ti. |
| 27 | ((Food or health$ or well being or wellbeing or wellness) adj Award$).ab,ti. |
| 28 | ((Food or nutrition) adj standard$).ab,ti. |
| 29 | Portion size$.ab,ti. |
| 30 | 12 or 13 or 14 or 15 or 16 or 17 or 18 or 19 or 20 or 21 or 22 or 23 or 24 or 25 or 26 or 27 or 28 or 29 |
| 31 | 11 and 30 |
| 32 | school$.ab,ti. |
| 33 | workplace$.ab,ti. |
| 34 | 32 or 33 |
| 35 | 31 not 34 |
| 36 | limit 35 to english language |
| 37 | limit 36 to humans |
| 38 | limit 37 to yr="1993 -Current" |
